# Supplementary material for: A Critical View of the Application of the APEX Software (Aqueous Photochemistry of Environmentally-Occurring Xenobiotics) to Predict Photoreaction Kinetics in Surface Freshwaters
Source: Molecules. 2019 Dec 18;25(1):9. doi: 10.3390/molecules25010009 (PMC7017383; doi:10.3390/molecules25010009)
Supplement: Supplementary file 1 [file molecules-25-00009-s001.zip › 3.2.4_gcc-4.4.0.zip!/3.2.4_gcc-4.4.0/3.2.4_gcc-4.4.0/doc/octave/html/interpreter/Error-Messages.html]

Error Messages - Untitled


Next: Format of Descriptions,
Previous: Printing Notation,
Up: Conventions


---

#### 1.3.4 Error Messages

Some examples signal errors. This normally displays an error message
on your terminal. Error messages are shown on a line beginning with
`error:`.

```
     fieldnames ([1, 2; 3, 4])
     error: fieldnames: wrong type argument `matrix'
```
